# A Case of Syndrome of Inappropriate Secretion of Antidiuretic Hormone Induced by Selpercatinib in a Patient With RET Fusion Gene‐Positive Non‐Small Cell Lung Cancer

**DOI:** 10.1002/rcr2.70352

**Published:** 2025-09-29

**Authors:** Yuma Tanaka, Naoya Ikegami, Kodai Miyamoto, Yusuke Shingu, Nobuhiro Okagaki, Hiroto Sakamoto, Tsukasa Nakanishi, Satoshi Nakamura, Kazuki Matsumura, Masakuni Ueyama, Yusuke Kaji, Seishu Hashimoto, Eisaku Tanaka, Yoshio Taguchi, Yuki Ohsumi, Tatsuo Nakagawa, Satoshi Matsunaga, Takashi Hajiro

**Affiliations:** ^1^ Department of Respiratory Medicine Tenri Hospital Tenri City Japan; ^2^ Department of Respiratory Medicine Kobe City Hospital Organization Kobe City Medical Center West Hospital Kobe City Japan; ^3^ Department of Respiratory Medicine Kurashiki Central Hospital Kurashiki City Japan; ^4^ Department of Thoracic Surgery Tenri Hospital Tenri City Japan; ^5^ Department of Endocrinology Tenri Hospital Tenri City Japan

**Keywords:** RET fusion‐positive non‐small cell lung cancer, selpercatinib, SIADH

## Abstract

An 80‐year‐old woman with a lung nodule in the right lower lobe and pleural thickening was diagnosed with lung adenocarcinoma by a surgical lung biopsy. The oncogene panel test showed a positive RET‐fusion gene mutation, and selpercatinib was administered as a first‐line treatment. She developed severe hyponatremia and was subsequently diagnosed with the syndrome of inappropriate secretion of antidiuretic hormone (SIADH). Discontinuing selpercatinib and initiating demethyltetracycline gradually improved SIADH. After confirming that sodium levels had returned to normal, we resumed and continued selpercatinib at a reduced dose without a recurrence of SIADH. This case suggests that selpercatinib could cause SIADH as an adverse event.

## Graphical abstract

We report a case of selpercatinib‐induced syndrome of inappropriate secretion of antidiuretic hormone (SIADH) that improved after drug withdrawal and was safely resumed at a reduced dose.

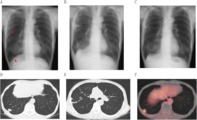

## 1 Introduction

In advanced or recurrent RET fusion gene‐positive non‐small cell lung cancer, selpercatinib is approved as a first‐line treatment [1]. Although hyponatremia has been reported in the LIBRETTO‐001 study, its aetiology and clinical course remain unclear [2]. We report a case of selpercatinib‐induced SIADH that improved after drug withdrawal and was safely resumed at a reduced dose.

## 2 Case Report

**FIGURE 1:**
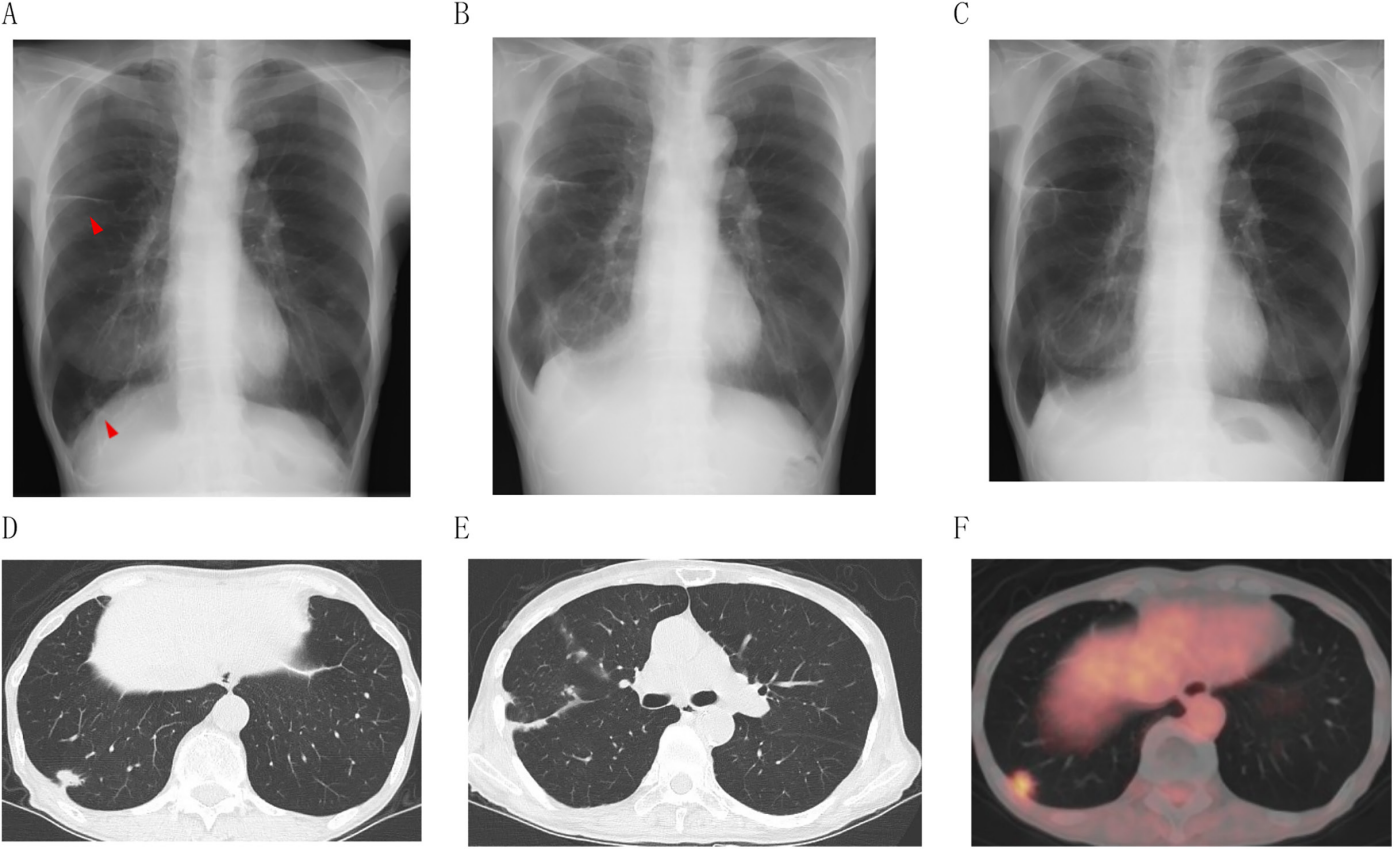
(A–C) Chest radiograph before surgery revealed thickening of the right minor fissure and a right lower lung field nodule (A), which had progressed before the start of selpercatinib (B), but improved 2 weeks after starting selpercatinib (C). (D, E) Chest computed tomography (CT) showed a nodule 17 mm in size in the right lower lobe (D) and multiple small nodules along the right interlobar pleura (E), suggesting pleural dissemination. (F) ^18^F‐FDG positron emission tomography (PET)‐CT showed elevated FDG uptakes in the right lower lobe nodule and multiple nodules on the interlobar pleura.

An 80‐year‐old woman was referred to our hospital for the evaluation of elevated serum CEA. She was thin, with a height of 152 cm and a weight of 33.7 kg. Chest computed tomography (CT) showed a nodule 17 mm in size in the right lower lobe and multiple small nodules along the right interlobar pleura, suggesting pleural dissemination (Figure 1). ^18^F‐FDG positron emission tomography (PET)‐CT showed elevated FDG uptakes in the right lower lobe nodule (SUV‐MAX 3.5) and multiple nodules on the interlobar pleura, without significant nodal or distant metastasis. An enhanced head magnetic resonance imaging (MRI) demonstrated no brain metastasis. A thoracoscopic partial lung resection and pleural biopsy confirmed lung adenocarcinoma (pathological T3N0M1a Stage IVA). RET fusion gene mutations were detected on the Oncomine Dx Target Test Multi‐CDx System. She was admitted for chemotherapy, and selpercatinib 160 mg twice daily was started the next day. The course of events following hospitalisation is shown in Table 1. On the 10th day of admission, a blood test showed a decrease in the sodium level to 115 mmol/L, and she suffered from dizziness. Test results are shown in Table 2 and Figure 1. Evidence of inappropriate AVP activity relative to plasma osmolality was observed in the absence of dehydration. She did not exhibit intracranial pituitary metastases, use diuretics, suffer from cardiac failure or have cirrhosis. Therefore, according to the diagnostic criteria [3], we diagnosed her with SIADH induced by selpercatinib. Selpercatinib was discontinued on the 11th day of admission. Fluid restriction and oral sodium chloride were started. Fludrocortisone was also added but was discontinued after 4 days because aldosterone secretion was not suppressed, ruling out mineralocorticoid‐responsive hyponatremia in the elderly. On the 15th day, demethyltetracycline was added. Hyponatremia had improved to Grade 2, and dizziness had improved on the 17th day. On the 46th day, the sodium level had improved to 137 mmol/L, and selpercatinib 40 mg once daily was resumed. A CT scan on the 73rd day showed the shrinkage of the pleural dissemination and the decrease in pleural effusion without new lesions. After selpercatinib was gradually increased to 80 mg twice daily, her serum CEA levels declined again. No worsening of hyponatremia was observed after the resumption and increase of selpercatinib.

**TABLE 1:** Clinical course and management.

Day	Event	Management in this case	General strategies
1	Selpercatinib 160 mg twice daily initiation		
3	Hypertension (Grade 2)	Amlodipine started	
10	Dizziness, hyponatremia, Grade 4 lymphopenia		
11	Persistent hyponatremia	Selpercatinib discontinued, fluid restriction (< 800 mL/day), NaCl 3 g/day, fludrocortisone added	Stop causative drug; fluid restriction; oral NaCl; consider loop diuretics, If acute or symptomatic, hypertonic saline can be administered
15	Persistent hyponatremia	demethyltetracycline 150 mg/day added fludrocortisone discontinued	If no response: vaptans, SGLT2 inhibitors, demethyltetracycline, urea in select cases
17	Serum sodium improved to Grade 2, dizziness improved	Continued demethyltetracycline, NaCl stopped	
20–40		Titration of demethyltetracycline up to 450 mg/day	
46	Selpercatinib resumed (40 mg once daily)		
73	CT: tumour shrinkage, decreased pleural effusion	Demethyltetracycline reduced to 300 mg/day	
Month 3–5	CEA levels rising	Selpercatinib gradually increased	
Month 5	Selpercatinib 80 mg twice daily		
Month 5~	CEA levels decreased		

Abbreviations: CEA, carcinoembryonic antigen; CT, computed tomography; Na, sodium; SGLT2; sodium‐glucose cotransporter 2.

**TABLE 2:** Laboratory data at the onset of hyponatremia.

Haematology			Biochemistry					
Hb	14.8	g/dL	BUN	11.0	mg/dL	ACTH	139	pg/mL
WBC	2350	/μL	Cr	0.39	mg/dL	Cortisol	31	μg/mL
Neut	63.9	%	Na	115	mmol/L	AVP‐ADH	5.2	pg/mL
Lymph	19.1	%	K	4.9	mmol/L	aldosterone	60	pg/mL
Mono	16.2	%	Cl	80	mmol/L	renin	< 0.2	ng/mL/h
Eo	0.4	%	UA	0.8	mg/dL			
Plt	11.1	×10^4^/μL	Osm	235	mosM/kg	Urine		
			Glu	131	mg/dL	UNa	28	mmol/L
			CRP	0.30	mg/dL	UK	34	mmoL/L
			FT4	1.58	ng/dL	UOsm	436	mosM/kg
			TSH	2.66	mIU/L	UCr	37	mmoL/L

Abbreviations: ACTH, adrenocorticotropic hormone; ADH, antidiuretic hormone; AVP, arginine vasopressin; BUN, blood urea nitrogen; Cr, creatinine; CRP, C‐reactive protein; Eo, eosinophil; FT4, free thyroxine; Glu, glucose; Hb, haemoglobin; Lymph, lymphocyte; Mono, monocyte; Na, sodium; Neut, neutrophil; Plt, platelet; TSH, thyroid‐stimulating hormone; UA, uric acid; UCr, urinary creatinine; UK, urinary potassium; UNa, urinary sodium; UOsm, urine osmolality; WBC, white blood cell.

## 3 Discussion

RET fusion gene is a driver gene mutation found in 1%–2% of non‐small cell lung cancers. For advanced or recurrent RET fusion gene‐positive non‐small cell lung cancer, selpercatinib is effective as a first‐line treatment [1]. In the LIBRETTO‐431 trial, the progression‐free survival (PFS) was significantly better in the selpercatinib group than in the platinum‐based chemotherapy (HR: 0.46, 95% CI 0.31–0.70, *p* < 0.001). Median 24.8 months versus 11.2 months. Adverse events reported in this study did not include hyponatremia [4]. In a long‐term follow‐up study of the LIBRETTO‐001 trial, hyponatremia was observed in 15.5% (Any Grade) and 9.9% (Grade 3 or higher) of patients [2]. However, as far as we have researched, details of cases of hyponatremia have not been reported in the literature, and the outcome of hyponatremia and whether selpercatinib can be continued remains unknown. This case suggests selpercatinib can induce SIADH, but treatment may continue after temporary discontinuation and dose reduction.

SIADH is a condition in which the antidiuretic effect of vasopressin persists despite hyponatremia [3]. Although SIADH can be caused by lung cancer itself, in this case, hyponatremia progressed after the start of selpercatinib, while chest x‐ray findings and serum CEA levels showed improvement of lung cancer (Figure 1). Serum CEA levels decreased from 86 ng/mL before treatment to 63 ng/mL on day 46. Since the treatment of the causative disease should lead to improvement of SIADH, lung cancer was considered unlikely to be the main cause of SIADH in our case. We were able to resume cancer treatment by reinitiating selpercatinib safely at a reduced dose. Even if SIADH occurred due to selpercatinib, dose reduction may enable continued treatment while maintaining its efficacy.

For treatment of chronic or asymptomatic SIADH, fluid restriction is performed, and if there is little improvement, vasopressin receptor antagonists, urea, SGLT2 inhibitors, demethyltetracycline and loop diuretics can be used [3]. In our case, SIADH occurred when selpercatinib was initiated at 160 mg twice daily in accordance with the protocol for the LIBRETTO‐431 trial [4]. Whether SIADH is induced dose‐dependently also by selpercatinib remains to be seen in future research. For the treatment of thyroid cancer in children aged 12 years or older, the dose of selpercatinib needs to be reduced according to the body surface area. Frail or low‐BMI patients may exhibit altered pharmacokinetics, suggesting the need for personalised dose adjustments to balance efficacy and safety.

The mechanism of selpercatinib‐induced SIADH remains unclear. As with osimertinib, which induces SIADH via off‐target intracranial kinase inhibition [5], selpercatinib could involve off‐target effects or altered renal sodium handling.

This is the first detailed report of hyponatremia as an adverse event caused by selpercatinib. This report has some limitations. As dynamic endocrine testing was not performed, lung cancer‐related hyponatremia cannot be ruled out and the pathophysiological mechanism of hyponatremia caused by selpercatinib is unknown. Pharmacovigilance registries or multicentre collaborations are needed to generalise our findings.

## Author Contributions

The first draft of the manuscript was written by Yuma Tanaka, and all the authors commented on previous versions of the manuscript. All the authors have read and approved the final manuscript.

## Consent

The authors declare that written informed consent was obtained for the publication of this manuscript and accompanying images using the consent form provided by the Journal.

## Conflicts of Interest

The authors declare no conflicts of interest.

## Data Availability

The data that support the findings of this study are available from the corresponding author upon reasonable request.
